# Leptin receptor signaling inhibits ovarian follicle development and egg laying in chicken hens

**DOI:** 10.1186/1477-7827-12-25

**Published:** 2014-03-20

**Authors:** Ming M Lei, Si Q Wu, Xiao W Li, Cong L Wang, Zhe Chen, Zhen D Shi

**Affiliations:** 1 Laboratory of Animal Breeding and Reproduction, Institute of Animal Science, Jiangsu Academy of Agricultural Sciences, Nanjing 210014, China; 2 College of Animal Sciences, South China Agricultural University, Guangzhou 510642, China

**Keywords:** Chicken leptin receptor, Extra-cellular domain, Antibody, Ovarian follicule development, Gene expression, Hens

## Abstract

**Background:**

Nutrition intake during growth strongly influences ovarian follicle development and egg laying in chicken hens, yet the underlying endocrine regulatory mechanism is still poorly understood. The relevant research progress is hindered by difficulties in detection of leptin gene and its expression in the chicken. However, a functional leptin receptor (LEPR) is present in the chicken which has been implicated to play a regulatory role in ovarian follicle development and egg laying. The present study targeted LEPR by immunizing against its extracellular domain (ECD), and examined the resultant ovarian follicle development and egg-laying rate in chicken hens.

**Methods:**

Hens that have been immunized four times with chicken LEPR ECD were assessed for their egg laying rate and feed intake, numbers of ovarian follicles, gene expression profiles, serum lipid parameters, as well as STAT3 signaling pathway.

**Results:**

Administrations of cLEPR ECD antigen resulted in marked reductions in laying rate that over time eventually recovered to the levels exhibited by the Control hens. Together with the decrease in egg laying rate, cLEPR-immunized hens also exhibited significant reductions in feed intake, plasma concentrations of glucose, triglyceride, high-density lipoprotein, and low-density lipoprotein. Parallelled by reductions in feed intake, mRNA gene expression levels of *AgRP, orexin*, and *NPY* were down regulated, but of *POMC*, *MC4R* and *lepR* up-regulated in Immunized hen hypothalamus. cLEPR-immunization also promoted expressions of apoptotic genes such as *caspase3* in theca and *fas* in granulosa layer, but severely depressed *IGF-I* expression in both theca and granulosa layers.

**Conclusions:**

Immunization against cLEPR ECD in egg-laying hens generated antibodies that mimic leptin bioactivity by enhancing leptin receptor transduction. This up-regulated apoptotic gene expression in ovarian follicles, negatively regulated the expression of genes that promote follicular development and hormone secretion, leading to follicle atresia and interruption of egg laying. The inhibition of progesterone secretion due to failure of follicle development also lowered feed intake. These results also demonstrate that immunization against cLEPR ECD may be utilized as a tool for studying bio-functions of cLEPR.

## Background

Leptin, secreted by the white adipocytes, plays important roles in regulating appetite, metabolism and energy homeostasis [1-3]. Leptin serves as a signal of the body fat content or energy reserve [4,5] for the brain to reduce food intake [6,7], and for the peripheral tissues to increase energy expenditure [1,8,9], and utilize the energy reserve and fat storage [10]. In addition, leptin is also instrumental to the initiation and the maintenance of the reproductive activities through its action in the hypothalamus to facilitate gonadotropin secretions [4,11-14]. Leptin has also been reported to directly antagonize ovarian estradiol and progesterone secretions stimulated by gonadotrophin follicle stimulating hormone (FSH) [15] or insulin-like growth factor I (IGF-I) [16,17], thereby inhibiting ovarian follicle development. Leptin has been reported to reduce cultured rat follicle growth speed and granulosa cell numbers [18], and decreased luteinizing hormone (LH) induced ovulations [19]. These inhibitory regulatory roles of leptin are conserved in chickens, as the ovarian follicle development is often perturbed in overweight hens [20,21], and that leptin has been observed to induce apoptosis of cultured chicken granulosa cells [22]. Nevertheless, much controversy exists over the presence of the chicken leptin gene or its protein product [3,23-26]. Difficulties in detecting leptin gene and products, together with the requirement for large quantities of leptin hormone in the case of administration studies, have hindered research into regulation of reproductive activities by leptin in chicken hens.

Fortunately, the leptin receptor (*lepR*) gene is present in the chicken genome [27,28] and its protein product, the leptin receptor protein (LEPR), functions to mediate hormone bioactivities [29]. For example, *in vivo* administrations of leptin from chicken and other species depressed chick feed intake [7], blood triglyceride concentrations [30], and accelerated reproductive development [31]. Furthermore, immunization against leptin stimulated abdominal fat deposition and feed intake [32]. These results of leptin administration and immunoneutralization should be mediated via LEPR signaling channels.

Since hormones fulfill their regulatory roles through the receptors, one could probe the signal transduction mechanism of the hormone by employing specific binding molecules that mimic hormonal actions [33-36]. Therefore, leptin-mediated regulation of the chicken ovarian follicular development could be studied by manipulating LEPR instead of using leptin directly. Previous studies have demonstrated that immunization against receptor extracellular domains (ECDs) could produce both anta- and agonistic effects [37], and that such a method could be adopted to study endocrine regulation of chicken ovarian follicular development [38]. The cLEPR possesses a long ECD, extending up to 810 amino acid residues [27]. Immunization against the domain proximal to the cell membrane has showed to mimic leptin signaling and reduced fat deposition in rats [unpublished data], and weight loss in chickens [unpublished data]. In the present study, we immunized the domain distal to the membrane, or the N-terminal domain of LEPR, to study its effects on ovarian follicle development in the laying hens, in terms of egg-laying performance, follicular atresia, gene expression and feed intake.

## Methods

### Obligatory ethical approval

The experiment was approved by the Research Committee of Jiangsu Academy of Agricultural Sciences and conducted with adherence to the Regulations for the Administration of Affairs Concerning Experimental Animals (Decree No.2 of the State Science and Technology Commission on November 14, 1988).

### Preparation of immunogens

A chicken LEPR ECD fusion protein, including a 36-residue leading sequence derived from the expression plasmid pRSETA (Invitrogen, Carlsbad, CA, USA), and the 200-residue sequence spanning from the N-terminal 101^st^ to 300^th^ amino acid residue of cLEPR, was expressed in E. coli strain BL21 (DE3). This cLEPR recombinant protein which was 236 amino acid residues long was purified to homogeneity by chromatography with Ni-NTA resin (Qiagen). The pure protein was then dissolved in a water /Grade 10 Injection white oil (Hangzhou Refinery, Hangzhou, China) mixture (in volume, 40% water and 60% oil) to reach a final concentrations of 1 and 2 mg/mL. The resulted emulsion was used as the immunogen in the experiment. A bovine serum albumin (BSA) immunogen was also prepared with the same method, with a final concentration of 1 mg/mL and 2 mg/mL.

### Animals and treatments

Sixty Yuehuang hens, 152-day old, were equally allocated into Control and cLEPR Immunized groups (each group n = 30). The hens were kept individually in battery cages, exposed to a 16 h light regime daily, and fed *ad libitum* a laying diet of 18% crude protein and 11.33 MJ/kg metabolizable energy. Egg laying was recorded daily at 17:00 and mean egg laying rate was calculated on a weekly basis. On day 1 of the experiment, the Immunized group was administered intramuscularly 1 ml cLEPR immunogen containing 1 mg of the recombinant cLEPR protein. Booster immunizations were performed on day 21, 63 and 91 with 1ml cLEPR immunogen containing 2 mg of the recombinant cLEPR protein. Similarly, Control hens were treated with 1 ml BSA immunogen on day 1 of the experiment and booster immunizations were performed on day 21, 63 and 91 with 1ml control immunogen containing 2 mg of the BSA. During the experiment, blood samples were collected, at 2 week intervals, by venipuncture of wing veins from 12 birds in each group, into a syringe containing 100 IU of heparin. The plasma was isolated by centrifugation at 1,000 *g* and stored at −20°C until analyzes until measurements of antibody titer. Blood serums collected on d 35 of the experiment were used for concentration measurements of glucose, triglycerides, cholesterol, high-density lipoprotein (HDL), low-density lipoprotein (LDL), and very low-density lipoprotein (VLDL).

On day 95, when egg laying started to decrease again in the Immunized cLEPR hens, all hens in each group were slaughtered. Ovarian follicles were collected and the numbers of large yellow follicles (LYF) and atretic follicles were recorded. From the largest five LYFs (F1 to F5) were granulosa and theca layers separated or isolated. The isolates were frozen in liquid nitrogen and subsequently stored under −70°C for gene expression analyses. Liver and adipose tissue samples also were collected, and snap frozen in liquid nitrogen.

### Measurement of blood anti-cLEPR antibody titer

A standard enzyme linked immunosorbent assay (ELISA) method was utilized to measure anti-cLEPR antibody titers in plasma. The recombinant cLEPR protein was used to coat the 96-well microtiter plates (5 g /well in 250 L). After blocking the plate with 1% skim milk and washing, 100 L of plasma sample, diluted 1:800 with 1% skimmed milk, was added to each well and incubated with anti-cLEPR antibody. The bound antibody was further labeled by addition of horse radish peroxydase (HRP)-conjugated sheep-anti-chicken antibody (Santa Cruz Biotechnology, CA, USA). Detection of the bound antibody was initiated by addition of chromogen tetraethyl benzidine (Sigma Chemical Co, St Louis, USA) solution containing 0.03% H_2_O_2_, and terminated with the addition of 2% H_2_SO_4_. Optical absorbance at 450 nm, representing anti-cLEPR antibody titer, was measured. To overcome assay bias between treatment groups, samples from each collection occasions were measured on the same plate.

### Western blotting

Liver tissue was homogenized and centrifuged. Approximately 30 μg of the supernatant was mixed with 2× Laemmli buffer (Sigma-Aldrich Corp., Saint Louis, MO), denatured, resolved on a denaturing PAGE, and transferred to a nitrocellulose membrane. The anti-stat3 and anti-beta-actin primary antibodies (1:200, Cell Signaling Technology, Inc., Boston, MA); and the anti-rabbit secondary antibody (1:50,000 Cell Signaling Technology, Inc., Boston, MA) were used for detection. The signal was developed using SuperSignal West-Pico kit (Thermo Fisher Scientific, Waltham, MA).

### Assays of serum glucose, triglycerides, cholesterol, HDL, LDL and VLDL

Concentrations of glucose, triglycerides, cholesterol, HDL, LDL, and VLDL were measured by using a ROCHE Modular P800 Automatic Biochemical Analyzer (Roche Diagnostics).

### Measurements of gene expressions

Real-time quantitative PCR was performed for quantification of *β-actin*, *agouti-related peptide* gene (*AgRP*), *neuropeptide Y* (*NPY*), *Proopiomelanocortin* (*POMC*), *Melanocortin 4 receptor (MC4R*), *orexin*, *lepR*, and *gonadotrophin- releasing hormone I* (*GnRH-I*) mRNA expression levels in the hypothalamus, *LHβ* and *FSHβ* in pituitary gland, *PPARγ* in the abdominal fat tissue and liver, *FSHR*, *LHR*, *IGF-I*, *fas*, *caspase3*, *bcl2*, *steroidogenic acute reguated protein* (*StAR*), *cytochrome P450, family 19, subfamily A, polypeptide 1*(*CYP19A1*), and *cytochrome P450, family 17, subfamily A, polypeptide 1* (*CYP17A1*) in ovarian granulosa and theca layers (Table 1). Total RNA was extracted from each tissue using Trizol (Invitrogen), and reverse transcribed to cDNA using ReverTra Ace qPCR RT Kit (Toyobo, Osaka, Japan). PCR reactions were carried out in a 50 μl reaction volume with SYBR Green I Master Mix reagent (Toyobo, Osaka, Japan) and 2.5 pmol specific primer pairs shown in Table 1. An ABI PRISM_7500 sequence detection system (Applied Biosystems, Foster City, CA, USA) was used to detect the amplification products. Upon completion of the real-time Q-PCR, threshold cycle (Ct, defined as the cycle at which a statistically significant increase in the magnitude of the signal generated by the PCR reaction was first detected) values were calculated by sequence detection software SDS Version 1.2.2 (Applied Biosystems). The levels of gene expression were expressed in the form of 2^-△△Ct^.

**Table 1:** Primers used in the real-time quantitative PCR of genes in chicken samples

**Gene**	**Accession number**	**Primer sequences (5′-3′)**	**Length(bp)**
*β-actin*	L08165	upstream: CCGAGAGAGAAATTGTGCGTGAC	166
		downstream: TCGGGGCACCTGAACCTCTC	
*FSHβ*	AY029204	upstream: GGCTGCGGTGACCATCCTGAATC	101
		downstream: GGCCCCAGTCCTCTCACAGTGCA	
*GnRH-I*	JN609557	upstream:TGTCCTCCTGTTCACCGCATCTG,	222
		downstream: TCGATCAGGCTTGCCATGGTTTC	
*LHβ*	HQ872606	upstream:GGGGGGAGCGCAGGTGTTG	220
		downstream: CCCGCAGGCCGTGGTGGT	
*AgRP*	AB029443	upstream: TCCCCTCGCCGCTGTGTC	137
		downstream: CATGGGAAGGTGGTGCTGATC	
*NPY*	NM_205473	upstream: GGAAAGAGATCAAGCCCAGAGAC	193
		downstream: ATGCACTGGGAATGACGCTATG	
*orexin*	AB056748	upstream: CGCTGGGCAAGAGGAAGAG	117
		downstream: GGCGCTCCTCACGTTTGC	
*MC4R*	AY545056	upstream: GCCAAGAACAAGAACCTCCATT	150
		downstream: TATGGTAAAGCTCTGTGCGTCTG	
*POMC*	NM_001031098	upstream: GGCCGAGGCACCCGTGTAC	141
		downstream: GCGGGGTGGTGGGGTGAC	
*LEPR*	NM_204323	upstream: TTTGCTGTTGGGCTTTCTTCAC	148
		downstream: AACCAGACCGGCTCCGTACA	
*FSHR*	NM_205079	upstream: TCCCACCAATGCCACAGAAC	155
		downstream: TGGGAAGGCTGGAAAACACA	
*LHR*	AB009283	upstream: GGGCATGAGCAACGAATCG	124
		downstream: CCGCCTGAGGTTTTTGTTGTC	
*IGF-I*	NM_001004384	upstream: TGCTGCTTTTGTGATTTCTTGAA	138
		downstream: AACCAGCTCAGCACCACACAGT	
*fas*	NM_001199487	upstream: TTCCCACACACACTGCACATAA	153
		downstream: CACACCGAGAAGAATTGCAGTAA	
*caspase3*	NM_204725	upstream: CCACGCTCAGGGGAAGATGTAT	173
		downstream: CGGTATCTCGGTGGAAGTTCTTA	
*bcl2*	NM_205339	upstream: CGCCGCTACCAGAGGGACTTC	192
		downstream: CGCCGCCGAACTCGAAGAAG	
*StAR*	NM_204686	upstream: GCCGGACGTGGGTAAGGTGT	184
		downstream: CGCCGTCTCGTGGGTGATC	
*CYP19A1*	NM_001001761	upstream: TGCCAGTTGCCACAGTGCCTATC	112
		downstream: GGCCCAATTCCCATGCAGTATC	
*CYP17A1*	NM_001001756	upstream: CGGGCAGCTTTCAGGCATG	189
		downstream: TGGCCATGATGTTGTGCACGTT	
*PPAR*	AB045597	upstream: GCTCCAGGATTGCCAAAGTG	137
		downstream: TCCCCACACACACGACATTCA	

### Statistical analysis

Differences between the Immunized and Control groups, in terms of anti-cLEPR ECD antibody titer, feed intake, the numbers of follicles, were analyzed by one-way analysis of variance. Differences of gene expression in hypothalamus, pituitary gland, the abdominal fat tissue, liver, and plasma concentrations of glucose, triglycerides, cholesterol, HDL, LDL, and VLDL were also analyzed by one-way analysis of variance. The means were compared by the LSD method. The data of gene expression in ovarian granulosa and theca layers were analyzed using the mixed model by SAS statistics software. The treatment group and various sizes of ovarian follicles were designated as fixed factors, while the hen identity was designated as the random factor. The means were compared with least squares means. All values are expressed as mean ± SEM. All statistical analyses were performed with SAS software Version 8.01 (SAS Institute Inc. Cary, NC. USA).

## Results

### Anti-cLEPR antibody titre

**Figure 1:**
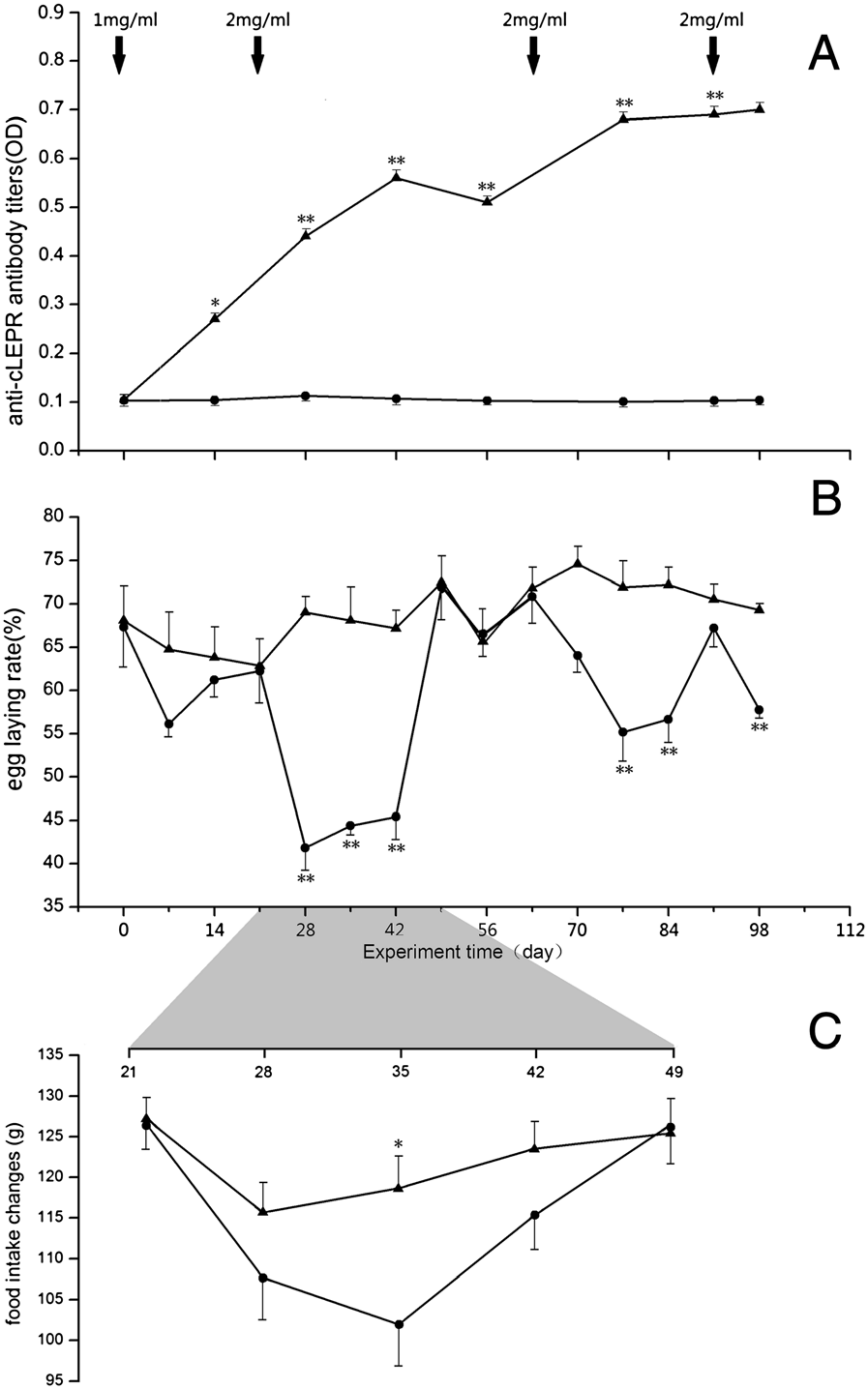
**Changes of anti-cLEPR antibody titer, egg laying rate, and feed intake between Control hens (****, n = 30) and cLEPR Immunized hens (****, n = 30). (A)** Changes of anti-cLEPR antibody titer in hen plasma **(B)** Changes of egg laying rate **(C)** Changes of feed intake. Vertical bars represent standard errors of the mean. Asterisks indicate significant differences (*:*P* < 0.05, **:*P* < 0.01). Arrows indicate administration of BSA and cLEPR immunogens and dose.

Antibody titers were measured by ELISA as optical density (OD) value at 450 nm. Throughout the experiment, the OD values were barely detectable in plasma samples of the Control hens, which were considered to be the non-specific binding of the assay. Fourteen days after the primary immunization, the anti-cLEPR antibody titer had already increased (*P* < 0.05) above the pre-immunized non-specific binding levels, and was further increased following the second or 1^st^ booster immunization (*P* < 0.01). The titers in the Immunized hens were maintained at high levels following the 2^nd^ and 3^rd^ booster immunizations on day 63 and 91, respectively (Figure 1A).

### Egg laying rate

Throughout the entire experimental period, the daily egg-laying rate in the Control hens remained relative stable, between 60% and 75%. However, in the Immunized hens, the rate started to decrease from 68% at the beginning of the experiment to approximately 55% around day 10 after the primary immunization (cLEPR dose 1 mg/ml) (Figure 1B). The rate subsequently increase to the level comparable to that in the Control group during the third week of the experiment (*P* > 0.05). However, the rate dropped sharply from the 4th week. After the first booster immunization (cLEPR dose 2 mg/ml), the egg laying rate went to a level below 45% and significantly lower (*P* < 0.01) than the rate in the Control hens. The laying rate remained depressed for further two weeks during the 5th and 6th week of the experiment, before rising back to control levels again at the 7th week of the experiment. The drops and rebounds of egg laying rate reappeared following the second booster cLEPR immunization, albeit with smaller magnitude and shorter duration (Figure 1B). Finally, the 4^th^ cLEPR immunization brought about a small drop of egg laying within five days before the hens were slaughtered (Figure 1B).

### Feed intake during egg laying drop

Measured during the expected egg laying decrease, following the first booster immunization, the feed intake of the Control group slightly decreased to 115 g in the first week, but thereafter gradually recovered to normal levels of 125 g in the following three weeks. The post-immunization drop of feed intake, however, was more marked in the Immunized hens, which, by day 35 of the experiment, was below 105 g. As the laying rate started to rebound after day 35, so did the feed intake, which recovered to the normal levels exhibited by the Control hens (Figure 1C). The difference in feed intake on day 35 was statistically significant between the Control group (118.66 ± 3.99 g) and Immunized group of chickens (101.97 ± 5.14 g, (P < 0.05)).

### Plasma metabolite concentrations

On day 35 of the experiment, i.e. when the egg laying rate and feed intake were minimal in the Immunized hens, after the first booster immunization, the plasma concentrations of triglycerides and HDL in Immunized cLEPR chickens were significantly lower than those in the Control chickens (*P* < 0.01) (Table 2), as were the concentrations of serum glucose and VLDL (*p* < 0.05). On the other hand, the concentrations of LDL in Immunized cLEPR chickens were significantly higher than in the Control chickens (*P* < 0.05).

**Table 2:** Concentrations of total glucose, triglycerides, cholesterol, HDL, LDL, and VLDL in hens (sera collected on d 14 after the first booster immunization, mean ± SEM, mmol/L, n = 24)

	**Blood glucose**	**triglyceride**	**cholesterol**	**HDL**	**LDL**	**VLDL**
*Controls*	14.088 ± 0.28^a^	17.1 ± 1.44^a^	4.91 ± 0.43^a^	2.1 ± 0.40^a^	0.83 ± 0.08^a^	1.99 ± 0.26^a^
*cLEPR-immunized*	12.97 ± 0.28^b^	12.07 ± 1.50^c^	4.02 ± 0.34^a^	1.19 ± 0.08^c^	1.43 ± 0.16^b^	1.39 ± 0.25^b^

Note: Means in the same column marked with different superscript letters differ significantly (^a-b:^*P* < 0.05; ^a-c:^*P* < 0.01).

### Ovarian follicle numbers

On day 5 after the 3^rd^ booster immunization with 2 mg of cLEPR immunogen, the Immunized hens had lower number of small yellow follicles (SYF) (*P* < 0.05), but greater number of atretic follicles (*P* < 0.05), compared to the Control hens (Table 3). Although the Immunized hens also had fewer numbers of LYF and large white follicle (LWF), compared to those of the Control hens, the differences were not statistically significant (*P* > 0.05).

**Table 3:** **Number of ovarian follicles counted on day 5 after the 4**^**st **^**administration (mean ± SEM,n = 8)**

**Types of follicles**	**Controls**	**Immunized**
*Large yellow follicles*	4.2 ± 2.0^a^	2.4 ± 2.5^a^
*Small yellow follicles*	8.4 ± 2.6^a^	4.8 ± 1.8^b^
*Large white follicles*	19.2 ± 9.3^a^	14.8 ± 8.0^a^
*Atretic follicle*	0 ± 0^a^	4.0 ± 0.9^b^

Note: Values in the same row with different superscript letters differ significantly (^a-b^, *P* < 0.05). Data are mean ± SEM (n = 8).

### Phosphorylated STAT3 of JAK2/STAT pathway

**Figure 2:**
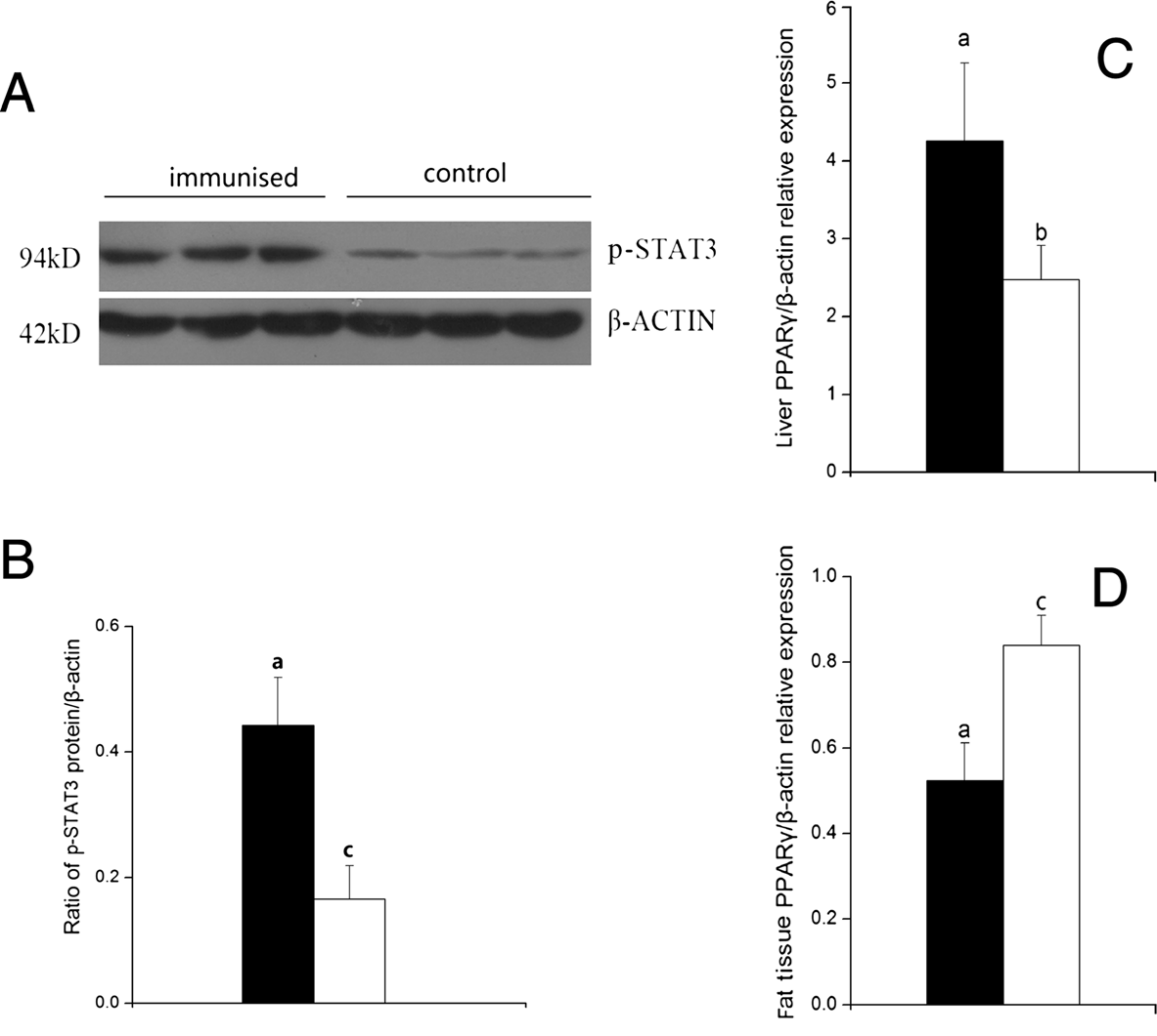
**Ratio of p-STAT3 protein/β-actin and levels of PPAR*γ *mRNA expression between Control hens (□, n = 8) and cLEPR Immunized hens (■, n = 8). (A)** Western blot of p-STAT3 (the band intensity was normalized to β-actin) **(B)** Ratio of p-STAT3 protein/β-actin (the ratios were used to analyze significant difference) **(C)***PPARγ* mRNA expression in liver **(D)***PPARγ* mRNA expression in abdominal fat tissues. Vertical bars represent the standard errors of the mean. Means not marked by a common letter are significantly different (a-b: *P* < 0.05; a-c: *P* < 0.01).

On day 5 after the 3^rd^ booster immunization, with 2 mg of cLEPR immunogen, the cLEPR Immunized groups had 2-fold increase of phosphorylated STAT3 proteins as quantified by the Western blot analysis in the liver tissue homogenate than in the Control group (Figure 2A and B) (*P* < 0.01).

### Gene expression

#### Follicle development regulating genes

**Figure 3:**
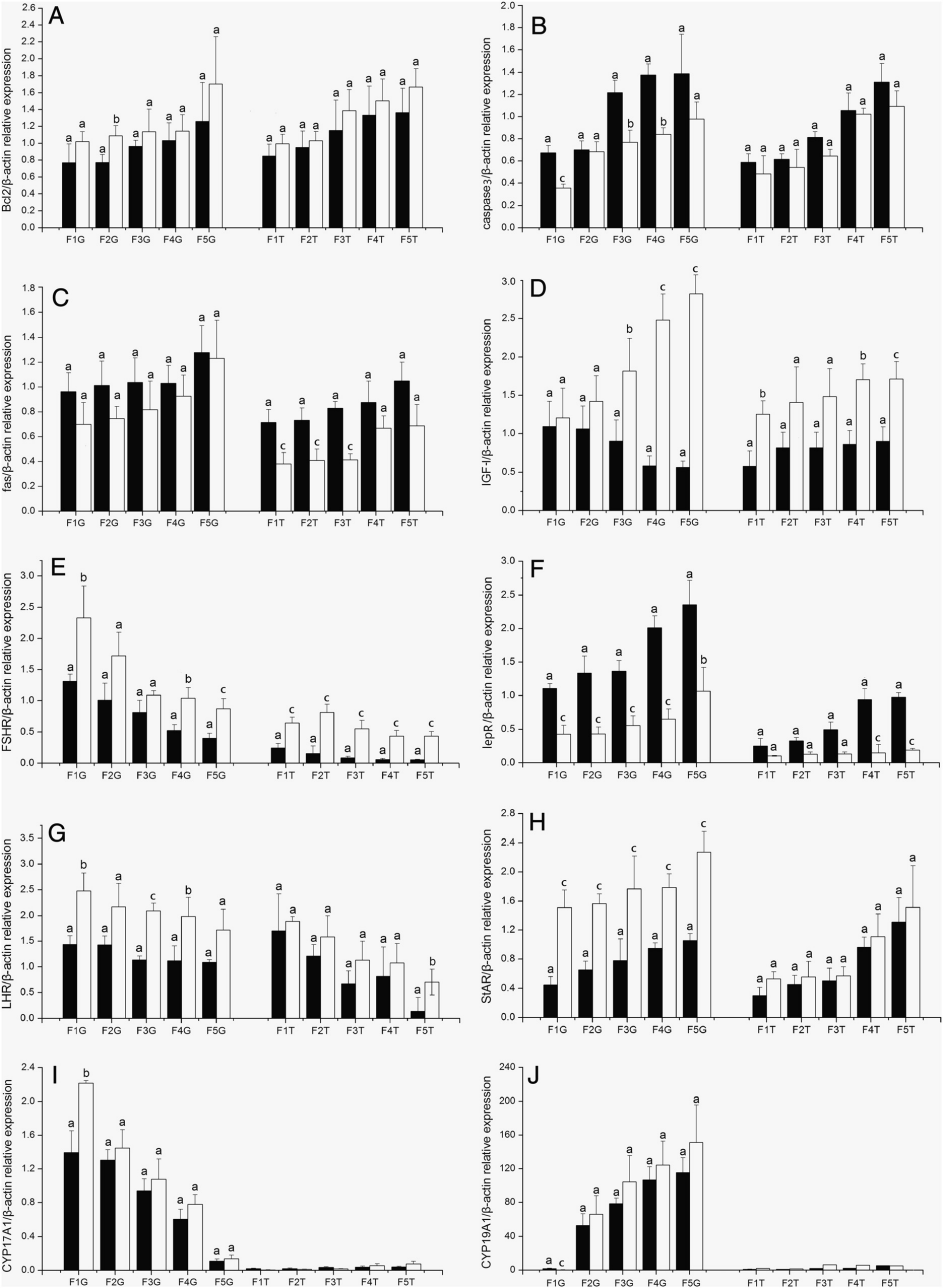
**The levels of mRNA expression relative to *β-actin *of ten genes in thegranulosa (G) and thecal (T) layer of various size ovarian follicles in Control hens (□, n = 8) and cLEPR Immunized hens (■, n = 8). (A)***bcl2***(B)***caspase3***(C)***fas***(D)***IGF-I***(E)***FSHR***(F)***lepR***(G)***LHR***(H)***StAR***(I)***CYP17A1***(J)***CYP19A1*. Fn represents the hierarchical order of follicle size, with the largest follicle designated as F1. Vertical bars represent the standard errors of the mean. Means not marked by a common letter are significantly different (a-b: *P* < 0.05; a-c: *P* < 0.01).

Following cLEPR immunization, the expressions of *lepR* and *caspase3* mRNA were strongly up- regulated in the granulosa layer in the F1 to F5 follicles (*P* < 0.01), while the expression level of *fas* was marginally but not statistically (*P >* 0.05) significantly up-regulated. However, *fas* expression was strongly up-regulated (*P* < 0.01) in the theca layer in F1 to F3 follicles. On the contrary, the expression of *bcl2* was slightly down-regulated in theca and granulosa layers, with more significant decrease in F5 and F2 follicles respectively (Figure 3).

The expressions of *LHR*, *StAR*, *FSHR* and *IGF-I* were down-regulated (*P* < 0.05) following cLEPR immunization in the granulosa layer in all class of follicles, and also in theca layer for the latter two genes. In the theca layer, *LHR* expression was significantly lower (*P* < 0.05) in F5 follicle, but its expression only dropped slightly in other larger follicles, in cLEPR immunized hens. In the cases of *CYP17A1* and *CYP19A1*, expression in granulosa layer was down-regulated in F1 follicles (*P* < 0.01 and *P* < 0.05 respectively).

#### Gonadotrophic hormone genes

**Figure 4:**
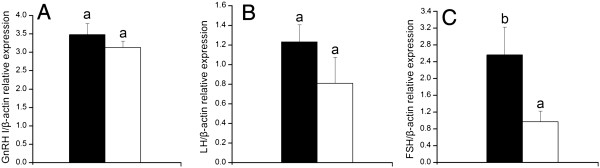
**The levels of mRNA expression relative to *β-actin *in hypothalamus and pituitary gland in Control hens (□, n = 8) and cLEPR Immunized hens (■, n = 8). (A)***GnRH-I* mRNA expression in hypothalamus **(B)***LHβ* mRNA expression in pituitary gland **(C)***FSHβ* mRNA expression in pituitary gland. Vertical bars represent the standard errors of the mean. Means not marked by a common letter are significantly different (a-b: *P* < 0.05).

The expressions of the *GnRH-I* gene in the hypothalamus and the *LHβ* gene (especially *FSHβ* (*P* < 0.05) gene) in pituitary gland were up-regulated following cLEPR immunization (Figure 4).

#### PPAR*γ* mRNA expression

The expression level of *PPARγ* mRNA were up-regulated (*P* < 0.05) in the liver, down-regulated (*P* < 0.01) in the abdominal fat tissues, following cLEPR immunization (Figure 2C and D).

#### Appetite regulating genes

**Figure 5:**
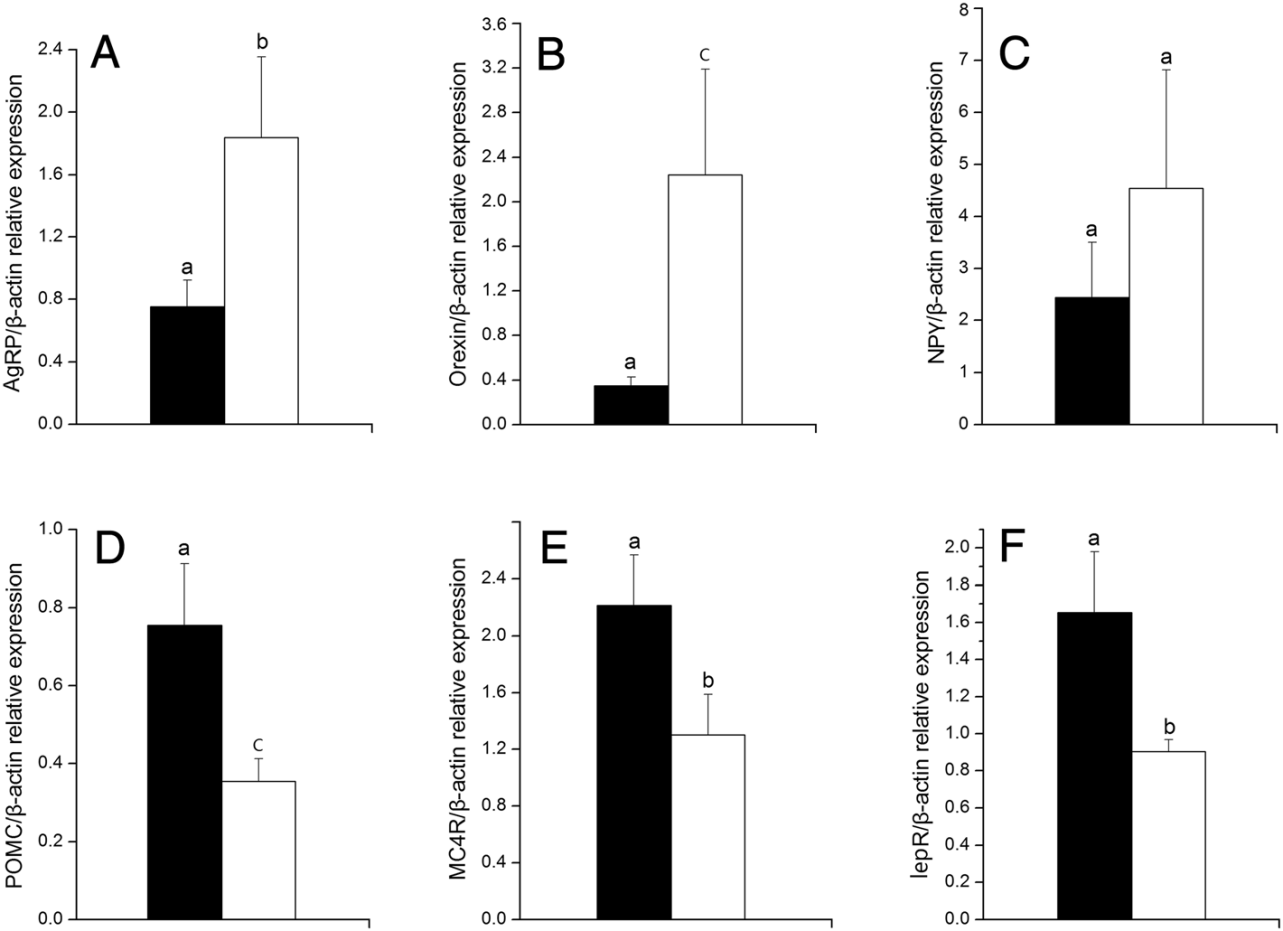
**mRNA expression levels relative to *β-actin *of six genes in hypothalamus in Control hens (□, n = 8) and cLEPR Immunized hens (■, n = 8). A)***AgRP* mRNA expression **(B)***orexin* mRNA expression **(C)***NPY* mRNA expression **(D)***POMC* mRNA expression **(E)***MC4R* mRNA expression **(F)***lepR* mRNA expression. Vertical bars represent the standard errors of the mean. Means not marked by a common letter are significantly different (a-b: *P* < 0.05; a-c: *P* < 0.01).

The expressions of the appetite-stimulating gene in the hypothalamus, *AgRP*, *orexin* were all down-regulated (*P* < 0.05) following cLEPR immunization. The expression of the anorexic genes, *lepR*, *POMC* and *MC4R*, however, were significantly up-regulated (Figure 5).

## Discussion

Through immunization approach, we have shown that generating anti-cLEPR ECD antibody caused ovarian follicle atresia by up-regulating the expression of apoptotic genes, and down-regulating the expression of pro-development genes, thus caused decreases in egg laying in chicken hens. These results indicate that manipulating leptin receptor activities using antibody allows the study of the regulatory roles of leptin in avian ovarian follicular development and egg laying.

In this study, the immunization approach was adopted for producing leptin receptor binding molecules. The anti- cLEPR antibodies generated appeared to enhance leptin signal transduction, as was demonstrated by the higher proportion of phosphorylated STAT3 protein, a key protein in leptin receptor signal transduction [27-29], in the liver tissues of cLEPR Immunized hens. This result accords to our unpublished observation of enhanced leptin receptor signaling in cLEPR Immunized rats. In addition, the expression level of *PPARγ* mRNA was down-regulated in adipose tissue in cLEPR-immunized hens. This effect agrees with the previous reports that administration of leptin down-regulated *PPARγ* mRNA expression in the adipose tissue [39,40]. The up-regulation of *PPARγ* mRNA expression in the liver of cLEPR-immunized hens also jibed with the up-regulation of *PPARγ* mRNA in cultured porcine adipose explants [40], which suggested a complex regulation of *PPARγ* by leptin. Nevertheless, these results demonstrated that anti- cLEPR antibody mimicked the bioaction of leptin to enhance LEPR signal transduction. In the present study, the antigen we prepared composed of the 200 (101^st^ to 300^th^) amino acid residues residing in the N-terminus CK-F3 domain of LEPR [41]. In two other studies with rats and chicken pullets, immunization against the sequence from 582^nd^ to 796^th^ amino acid residues of LEPR, in the F3-F3-F3 domain proximal to the cellular membrane, also enhanced LEPR signaling, reduced adipose tissue deposition [unpublished data], reduced live weight, while increased feed intake [unpublished data]. Results of our study and the above results indicate that antibodies bound to LEPR could trigger a signal transduction that stimulated metabolism. It appears that antibodies directed against many epitopes of LEPR ECD could trigger a receptor signal transduction. This may be due to the nature of LEPR, which exists as a dimer [42,43], hence the binding of any molecule, including antibody, to the dimer would lead to the formation of a molecule trimer that will cause receptor signal transduction [41,43].

Leptin receptor that mediates leptin bioactivity is widely expressed by various tissues [44-46]. Our results showed that *lepR* is expressed in both granulosa and theca layers, with the expression level being higher in granulosa than in theca tissue. These results were consistent with the results previous reported by Cassy et al. [47]. In both studies, the *lepR* expression level decreases as the follicle enlarges, which might suggest a weaker expression of *lepR* in large more mature follicles diminished the negative regulatory role of leptin [47]. This form of regulation could be compared to the direct inhibition by leptin of gonadotropin and growth factor stimulated steroid hormone production by cultured rat granulosa cells [15-17]. Moreover, the expression of ovarian *lepR* was up-regulated by *ad libitum* feeding of the breeder hens [47], which increased plasma leptin concentrations [23]. Likewise, our study showed up-regulation by immunization against cLEPR ECD. In many circumstances, cytokine hormones such as GH and leptin could up-regulate gene expression of its own receptors [48,49], thus results from our study and those from previous studies suggest that an up-regulation of *lepR* expression in ovarian tissues by *ad libitum* feeding and overweight hens was caused also by a molecule directed to *lepR*, which could be leptin or a molecule analogous to it, a possibility which is beyond the scope of the present study.

Both the *ad libitum* feeding [47] and immunization caused an up-regulation of *lepR* which was more pronounced in smaller F4 or F5 follicles. Thus the *lepR* mediated negative regulation of ovarian follicular development could be stronger in these small LYFs, or in even smaller follicles such as SYF and LWF. As expected, upon being slaughtered on Day 5 after the 4^th^ cLEPR immunization, severe atresia of SYFs occurred, causing a significant decrease to the number of healthy SYFs compared to the Control hens. Though the number of LYFs and the daily egg laying rate was only slightly decreased shortly for 5 days, after the 4^th^ cLEPR immunization, atresia of LYFs was also observed following longer lag by about Day 10 after administration of LEPR (unpublished observations). Therefore the negative regulation by anti-cLEPR antibody first appeared in SYFs, and then gradually extended to LYFs as the antibody titers continued to rise following each administration of antigen, even though the more mature larger LYFs were more resistant to leptin attenuation. This effect by anti-cLEPR antibody was also reflected by the stronger down-regulations of *IGF-I* and *FSHR*, but up-regulation of *lepR* in smaller than in larger LYFs. The ultimate effect is the decrease in egg laying rate in cLEPR Immunized hens. There also existed a dose dependent effect of anti-cLEPR antibody titer on reduction of laying rate between the primary and first booster immunizations. However laying rate also recovered to the level in the control hens after the initial drop when the antibody titers were still high, as was seen prior to the 2^rd^ and 3^th^ booster immunizations. Further, the higher titer after the 2^nd^ booster immunization was associated with a less degree of drop in egg laying rate, compared with the situation after the 1^st^ booster immunization. It is currently unknown whether the developing follicles could become refractory to persistent antibody stimulation, and still developed to ovulation. The other explanation is that more antibodies could be generated towards the ‘foreign’ leading peptide derived from the expression vector, instead of to the ‘self’ cLEPR domain in the recombinant antigen used. Antibodies to the leading peptide would not bind to cLEPR and not interfere ovarian follicle development. In this study, the number of LWFs was not affected by cLEPR immunization, which may suggest the development and function of LWFs may be free from leptin regulation. This assumption was supported by the finding that gonadotrophin receptor was not expressed in these class of follicles [50,51], and that the leptin effect was mediated together with gonadotrophin and growth factor regulators [15-17]. The interruption of egg laying following cLEPR immunization is comparable to the reduced or even erratic egg laying in *ad libitum* fed, overweight fast growing breeder hens [20,21]. In the latter, there was an overgrowth of ovarian follicles, resulting in more than one SYFs recruited together into the hierarchical development during each ovulation/oviposition cycle [52,53], which results in presence of an extraordinary number of LYFs, a phenomenon akin to the polycystic ovary syndrome in obese humans [54]. However, despite of the overgrowth, the LYFs in overweight hens were never as large as those in restrictedly fed low weight hens, nor as high as steroid hormone production per follicle [20]. These could indicate that the largest follicles were not as mature as those of restrictedly fed low weight hens, and were not able to secrete sufficient steroid hormones to trigger pre-ovulatory LH surge or could not respond to it, thus leading to a reduced egg laying rate [21]. In addition, despite the overgrowth, follicle atresia was often observed especially at SYFs and small LYFs [53]. This phenomenon again indicated that some metabolic or endocrine factors associated with high nutrition intake and overweight might induce cell apoptosis and atresia of the small follicles, similar to the atresia caused by anti-LEPR antibody.

The anti-LEPR antibody is not able to cross the blood–brain barrier, therefore cLEPR immunization does not affect hypothalamic *GnRH I* and *LH* gene expressions in pituitary gland. On the contrary, a lack of negative feedback hormone regulation by oestrogen and inhibin due to the atresia of SYFs and especially LYFs was observed. The gene expression of *FSH* became significantly up-regulated in the pituitary gland. These results indicated that the anti-cLEPR antibody did not impair hypothalamic-pituitary functionality over reproductive function, and perturbation of the egg laying should reside at ovarian follicles. The atresia of SYFs and most probably LYFs was associated with up-regulation of apoptotic genes *caspase3* and *fas*, and down- regulation of anti-apoptotic genes *bcl2* and *IGF-I*. Except from the case of *fas*, the effect of immunization against cLEPR was more severe, especially for *IGF-I*, on smaller LYFs, F4 and F5, and possibly even SYFs, whose expressions were not analyzed. These results also indicate the atretic effects of anti-cLEPR antibodies have exerted first to SYFs and smaller LYFs. Previously, Sirotkin and Grossmann had shown that low and moderate levels of leptin inhibited expressions of apoptotic genes and stimulated expression of anti-apoptotic gene *bcl2* in cultured chicken ovarian follicle tissue [22]. However the reverse was true when leptin concentration in the culture medium was further increased [22]. It seemed that the anti-cLEPR antibody generated following immunization in this study had mimicked an effect created by high levels of leptin concentration. This further explains why only low numbers of SYFs and LYFs undergo atresia in *ad libitum* fed overweight hens, whose leptin levels could be very low and any effect could be mild compared with the high antibody titer in this study. Besides, low level of leptin stimulated, while high level decreased the release of progesterone and estradiol by cultured ovarian follicular tissue [22]. This phenomenon was parallelled by the decrease in the expressions of *LHR*, *FSHR*, *StAR* and *CYP17A1* in our study. These results further indicated that gonadotropin hormone receptor response and steroid hormone secretion competence were reduced by the anti-cLEPR antibody affected cells and follicles.

The rapid growth of LYFs during hierarchical growth occurs with the deposition of large amount of yolk lipids and the associated proteins vitellogenin and very low density lipoprotein [55]. When follicular growth is interrupted, so are the synthesis and secretion of progesterone. In the mammals, a high circulating concentration of progesterone during the late pregnancy functions to induce a state of leptin resistance, so to further enhance food intake even when a positive energy balance is already reached [56-58]. In our study, such effect was expressed as a decrease in feed intake, when laying drop and follicular atresia occurred after cLEPR immunization, or when the ovarian follicles stopped secreting progesterone. The plasma concentrations of HDL in cLEPR Immunized chickens were significantly lower than those in the Control chickens. The plasma concentration of LDL in Immunized cLEPR chickens was significantly higher than in the Control chickens. These results accord with the results previous reported by Maki [59]. In addition, blood concentrations of glucose and triglycerides in the normal laying Controls hens were significantly higher than those in the Immunized hens. These results also suggest a state of ‘leptin resistance’ in the normal laying Control hens. Both a reduction of hypothalamic expression of *Ob-Rb* and an increase in plasma leptin binding protein were implicated to fulfill the leptin resistance effect of progesterone [58]. The results of this study favored the latter theory: because the *lepR* expression in hypothalamus was increased, yet expressions of the orexigenic genes, *Orexin*, *NPY* and *AgRP* were down regulated, and those of anorexigenic genes, *MC4R* and *POMC* up-regulated. These results were in direct contrast to the up-regulation of orexigenic genes, but down regulation of anorexigenic genes in LEPR immunized growing rats [unpubilished data] and also chicken pullets [unpublished data]. In these growing animals free from progesterone interference, the anti-cLEPR antibody stimulated metabolism, decreased fat deposition and even body weight, as well as leptin secretion, thus stimulated appetite and feed intake. Results from our study demonstrate the importance of progesterone in regulation of feed intake in the laying hens.

## Conclusions

Immunizing against LEPR ECD in hens generated antibodies that mimic leptin bioactivity by enhancing LEPR signal transduction. This regulated apoptotic gene expression in ovarian follicles, down-regulated the expression of genes supporting follicular development and hormone secretion, leading to follicle atresia and interruption of egg laying. Inhibition of progesterone secretion due to failure of follicle development also lowered feed intake. The results of this study demonstrate immunization against LEPR ECD may be utilized as a tool for studying bio-functions of chicken LEPR.

## Abbreviations

AgRP: Agouti-related peptide gene; BSA: Bovine serum albumin; CYP17A1: Cytochrome P450, family 17, subfamily A, polypeptide 1; CYP19A1: Cytochrome P450, family 19, subfamily A, polypeptide 1; ECD: Extra-cellular domain; ELISA: Enzyme linked immunosorbent assay; FSH: Follicle stimulating hormone; FSHR: Follicle stimulating hormone receptor; HDL: High-density lipoprotein; IGF-I: Insulin-like growth factor 1; LDL: Low-density lipoprotein; LEPR: Leptin receptor; LH: Luteinizing hormone; LHR: Luteinizing hormone receptor; LWF: Large white follicle; LYF: Large yellow follicles; MC4R: Melanocortin 4 receptor; NPY: Neuropeptide Y; POMC: Proopiomelanocortin; PPARγ: Peroxisome proliferator-activated receptor gammar; StAR: Steroidogenic acute regulated protein; SYF: Small yellow follicle; VLDL: Very low-density lipoprotein.

## Competing interests

The authors declare that they have no competing interests.

## Authors’ contributions

MML and ZDS designed the study, while XWL and CLW constructed the LEPR ECD protein. MML and SQW carried out the animal experiment, collected and analyzed the samples. MML and ZDS prepared the manuscript with correction input by ZC. All authors read and approved the final manuscript.
